# Testing for sexually transmitted infections in general practice: cross-sectional study

**DOI:** 10.1186/1471-2458-10-667

**Published:** 2010-11-03

**Authors:** Katharine E Sadler, Nicola Low, Catherine H Mercer, Lorna J Sutcliffe, M Amir Islam, Shuja Shafi, Gary M Brook, Helen Maguire, Patrick J Horner, Jackie A Cassell

**Affiliations:** 1 Research Department of Infection and Population Health, University College London, London, UK; 2 Institute of Social and Preventive Medicine, University of Bern, Bern, Switzerland; 3 Health Protection Agency, Northwick Park Microbiology Laboratory, London, UK; 4 Central Middlesex Hospital, North West London Hospitals Trust, Acton Lane, Park Royal, London, UK; 5 Health Protection Agency, London Region, London, UK; 6 St Georges Hospital Medical School, London, UK; 7 Department of Social Medicine, University of Bristol, Bristol, UK; 8 Brighton and Sussex Medical School, University of Brighton, Falmer, Brighton, UK

## Abstract

**Background:**

Primary care is an important provider of sexual health care in England. We sought to explore the extent of testing for chlamydia and HIV in general practice and its relation to associated measures of sexual health in two contrasting geographical settings.

**Methods:**

We analysed chlamydia and HIV testing data from 64 general practices and one genitourinary medicine (GUM) clinic in Brent (from mid-2003 to mid-2006) and 143 general practices and two GUM clinics in Avon (2004). We examined associations between practice testing status, practice characteristics and hypothesised markers of population need (area level teenage conception rates and Index of Multiple Deprivation, IMD scores).

**Results:**

No HIV or chlamydia testing was done in 19% (12/64) of general practices in Brent, compared to 2.1% (3/143) in Avon. In Brent, the mean age of general practitioners (GPs) in Brent practices that tested for chlamydia or HIV was lower than in those that had not conducted testing. Practices where no HIV testing was done had slightly higher local teenage conception rates (median 23.5 vs. 17.4/1000 women aged 15-44, p = 0.07) and served more deprived areas (median IMD score 27.1 vs. 21.8, p = 0.05). Mean yearly chlamydia and HIV testing rates, in practices that did test were 33.2 and 0.6 (per 1000 patients aged 15-44 years) in Brent, and 34.1 and 10.3 in Avon, respectively. In Brent practices only 20% of chlamydia tests were conducted in patients aged under 25 years, compared with 39% in Avon.

**Conclusions:**

There are substantial geographical differences in the intensity of chlamydia and HIV testing in general practice. Interventions to facilitate sexually transmitted infection and HIV testing in general practice are needed to improve access to effective sexual health care. The use of routinely-collected laboratory, practice-level and demographic data for monitoring sexual health service provision and informing service planning should be more widely evaluated.

## Background

Enhancement of sexual health services in the primary care setting has been an objective of health policy in England since the publication of the National Strategy for Sexual Health in 2001 [1], and confirmed in later national strategic plans [2]. Improving the availability of testing for sexually transmitted infections (STI) and HIV has considerable potential to improve sexual health in the UK, given that around a third of those attending specialist genitourinary medicine (GUM) clinics for suspected STI have already attended general practice [3]. Two large-scale studies in England have shown the potential capacity of primary care to contribute to chlamydia screening and management [4,5]. In terms of HIV testing, only a small proportion of HIV tests were undertaken in primary care in the early 1990s [6], but the establishment of antenatal HIV screening and new guidelines aimed at achieving wider coverage of HIV testing have emphasised the key role of primary care in making early diagnoses to minimise the high proportion of avoidable deaths still seen among those diagnosed late [7-9].

There are still substantial challenges for primary care in implementing STI and HIV control, especially for populations that are not specifically targeted by screening programmes. Rates of testing for genital chlamydial infection, the most commonly reported bacterial STI, vary considerably between general practices, even within a locality [10,11]. A national probability survey in 2000 found that general practices accounted for one quarter of voluntary confidential HIV tests (VCT) among those reporting testing in the past 5 years [6]. Testing for the two infections might not, however, go hand in hand; in one study women diagnosed with chlamydial infection in general practice were much less likely to have been tested for HIV than those diagnosed in GUM clinics [12]. Monitoring STI control activities in general practice is difficult, however, because data are not yet routinely collected either for either HIV or chlamydia testing in primary care, apart from the special cases of antenatal HIV screening and chlamydia tests taken in under 25 year olds in practices participating in a chlamydia screening programme in England [13].

The aim of this study was to determine how routinely available data can be used to explore patterns of chlamydia and HIV testing and their relation to practice and population characteristics in general practices. The study formed the initial phase of a larger project aimed at developing and targeting an intervention to support the management of STI in primary care. Our specific objectives were: to determine chlamydia and HIV testing rates; to determine the proportion of patients registered with general practices that had undertaken chlamydia and HIV testing; and to examine associations between HIV or chlamydia testing rates and population measures of teenage pregnancy and socioeconomic deprivation.

## Methods

### Study populations

We chose to focus our study on two contrasting UK populations with differing health service configurations: Brent, in London and Avon, in the South West of England. The demographic characteristics of the populations are typical of those of many young UK residents. We studied the populations of Brent, in London (total population 278,560) and Avon, in the South West of England (total population 984,000). The population of Brent is ethnically diverse; 55% are from non-white ethnic groups compared to under 10% in Avon [14]. Brent is in an urban area served by one primary care trust (PCT), one GUM clinic and 71 general practices. Avon has urban suburban and rural areas served, at the time, by five PCTs, two GUM clinics and 143 general practices. HIV prevalence in 2007 was 3.76/1000 in Brent and 1.53/1000 in Bristol PCT [15].

### Study design

We did a cross-sectional study using laboratory data about chlamydia and HIV tests in Brent and Avon. In Brent, we analysed three years of data collected between 1 July 2003 and 30 June 2006 from the microbiology laboratory at Northwick Park Hospital, which serves the GUM clinic and 64 of the 71 general practices in this area. In Avon, we used a database containing data on tests and results for laboratory-diagnosed STIs from all general practices and the area's two largest GUM clinics in 2004 (the only year for which comparative data were available) [11]. In both areas we counted individuals who had been tested only once, even if they had had multiple specimens taken. We excluded HIV tests reported from antenatal clinics because these should be taken unless a woman specifically declines to be tested and are separately audited [16]. We also restricted the analysis of chlamydia tests to those taken outside of England's recently established National Chlamydia Screening Programme (NCSP) since Avon only entered the programme in 2007 while Brent joined in 2004 [13]. This exclusion is unlikely to bias our analyses as the number of NCSP chlamydia tests carried out was small in both areas: only 42 NCSP tests were undertaken in Avon during the first 6 months, while 1,155 NCSP tests were undertaken (mainly from contraception clinics) in Brent in the year to March 2006 [17].

We used data from the National Primary Care Database (NPCD) [18] to obtain the number of patients registered with each general practice. We then applied the numbers of chlamydia and HIV tests obtained from the laboratory records to calculate testing rates for each infection (per 1000 practice population aged 15-44 years). Each practice was coded as to whether or not specimens for chlamydia or HIV had been taken during the study time period.

Practices were then mapped to their ward and super-output area (SOA, geographic areas of typical population size 1500) [19], using data from the National Health Service Postcode Directory. Ward level teenage conception rates (per 1000 female population aged 15-19 years) were obtained from PCTs; and published SOA level Index of Multiple Deprivation (IMD) scores assigned [20]. The IMD score is the average score based on six weighted domains - income, employment, health deprivation and disability, education, skills and training, barriers to housing and services, crime and living environment; an increasing score indicates increasing deprivation.

We linked these data with general practitioner (GP) characteristics (age and sex provided by NPCD, which exclude temporarily employed GPs), from which we calculated the ratio of all principal and all female GPs per 1000 female patients for each practice. The mean age of all GPs per practice was calculated using mid-point estimates of five aggregate age-groups: GPs aged under 30 years, 31-40 years, 41-50 years, 51-60 years and 61+ years. Using the mean age of all GPs (52.4 years) as a mid-point, the proportion of GPs per practice aged under 51 years (the first three age-groups) was also calculated for Brent. This was not possible for Avon where the proportion of full time GPs aged under and over 50 was calculated instead.

We used descriptive statistics to examine chlamydia and HIV testing patterns in general practices and GUM clinics. We used the Mann-Whitney U test (for medians), chi-squared test (for proportions) and the t-test (for means) to examine differences between those general practices that had and had not undertaken chlamydia or HIV tests. Detailed data about chlamydia testing in Avon have been presented elsewhere and are not repeated here [11].

All analyses were carried out using STATA version 8.2 (Stata Corporation, College Station, Texas, USA). The study was approved by the South-West Multicentre Research Ethics Committee.

## Results

### General practice and GUM clinic contributions to chlamydia and HIV testing

Between 2003-2006, a total of 9517 chlamydia tests and 2743 HIV tests were taken in 64 general practices in Brent PCT. More tests for both chlamydia and HIV were taken in the GUM clinic than in all general practices combined; the ratio of chlamydia tests in general practice compared to GUM was 0.6:1 (9517 and 16762 tests respectively) while the HIV testing ratio was 0.2:1 (2743 and 13893 tests respectively). In contrast in Avon, the general practice:GUM chlamydia and HIV testing ratios in 2004 were higher at 1.2:1 (15802:12962) and 0.6:1 (4881:8419), respectively.

### Distribution of chlamydia and HIV tests by age and sex

**Figure 1:**
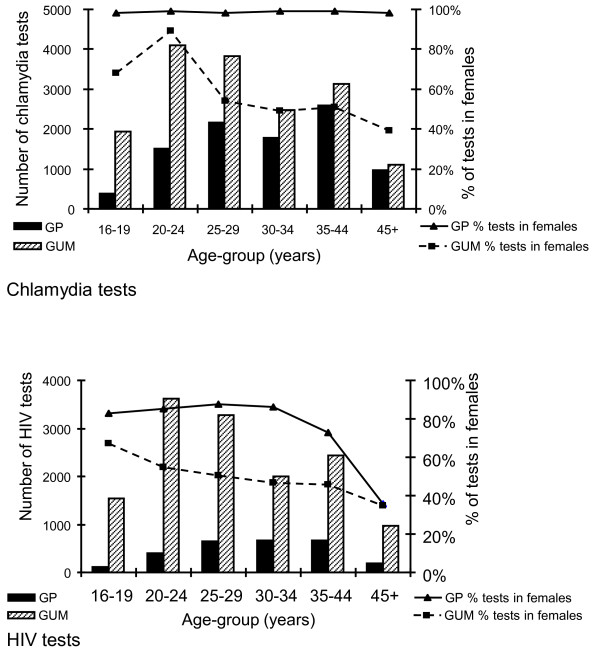
**Age and Sex distribution of chlamydia and HIV tests taken in genitourinary medicine and general practices in Brent primary care trust, mid-2003 to mid-2006**.

In Brent's general practices, only 20% (1905/9423) of chlamydia tests were carried out in patients aged under 25 years, compared with 36% (6024/16583) of tests done in its GUM clinic (*p *< 0.001) and 39% (6138/15802) of tests done by Avon's general practices (*p *< 0.001) [Tables 1 and 2]. In Brent general practices, females accounted for 98% (9285/9474) of chlamydia tests and 79% (2152/2732) of HIV tests, compared with 55% (9175/16741) and 51% (7114/13883) respectively in the GUM clinic (p < 0.001) (Figure 1). In Avon's general practices, females accounted for a smaller proportion of testing: 85% (13496/15802) of chlamydia tests and 77% (3754/4881) of HIV tests. Likewise, in Avon's GUM clinics females accounted for a smaller proportion of testing: 43% (5522/12936) of chlamydia tests and 42% (3520/8403) of HIV tests (denominators differ slightly because of missing data on sex for 26 chlamydia and 16 HIV tests).

**Table 1:** Characteristics of general practices in Brent, according to chlamydia and HIV testing practice

**Practice Characteristic**	**Practices that had tested for *Chlamydia trachomatis *(n = 48)**	**Practices that had not tested for *Chlamydia trachomatis *(n = 16)**	**p-value for difference**	**Practices that tested for HIV (n = 30)**	**Practices that had not tested for HIV (n = 34)**	**p-value for difference**
Median list size (range)	5351 (1091-14,128)	3354 (2080-7000)	0.04	4361 (2494-14,128)	4037 (1091-10,278)	0.25
Median IMD score	24.6	26.7	0.33	21.8	27.1	0.05
Median teen conception rate	21.6	23.5	0.08	17.4	23.5	0.07
Ratio of female GPs per 1000 female practice population (range)	0.56 (0-1.54)	0.42 (0-1.01)	0.38	0.51 (0-1.54)	0.55 (0-1.35)	0.72
Ratio of all GPs per 1000 female practice population (range)	1.1 (0.63-3.39)	1.03 (0.45-1.47)	0.73	1.2 (0.45-3.39)	1.02 (0.47-2.36)	0.42
Mean age of GPs (years)	50.8	57.4	0.002	50.1	54.5	0.02
*% GPs < = 51 years*	*51.1*	*14.8*	-	*48.8*	*40.5*	-
*% GPs >51 years*	*48.9*	*85.2*	-	*51.2*	*59.5*	-

### Intensity of chlamydia and HIV testing in general practices

Among the 64 general practices in Brent, there were marked differences in testing for chlamydia and HIV over the three year study period; 56% (n = 34) of practices had taken no specimens for HIV testing, 25% (n = 16) had taken no specimens for chlamydia testing, and 19% (n = 12) had not taken specimens for either infection. There was no evidence of changing testing rates over the study period. In 143 general practices in Avon in 2004, by contrast, only 2% (n = 3) had not taken any specimens for HIV tests and 10% (n = 14) had not taken any specimens for chlamydia testing.

**Figure 2:**
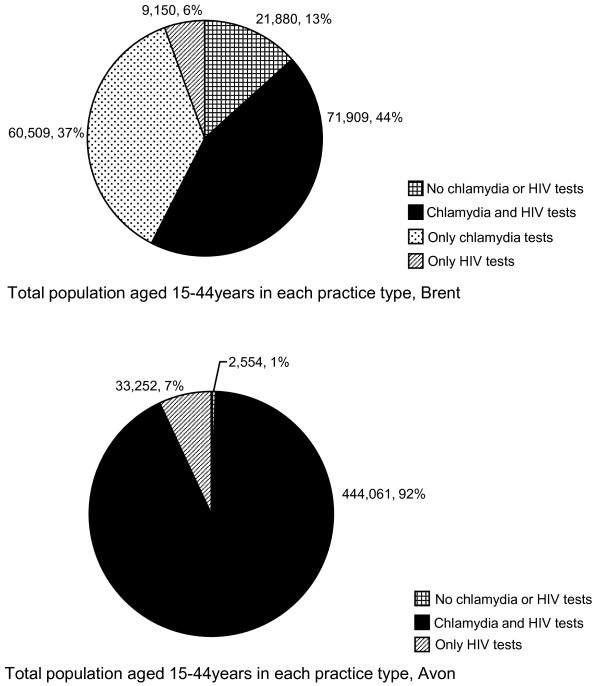
**The distribution of the 15-44 year old population by general practice testing status in Brent and Avon**.

Figure 2 shows the distribution of the registered population aged 15-44 by the testing status of their practice in both areas. In Brent, 44% of the population belonged to a practice which had tested for both chlamydia and HIV, while this proportion was 92% in Avon.

Among practices that had tested for chlamydia, the mean annual testing rates were 33.2 (range 0.6 to 212.6) and 34.1 (range 0.5-82.4) per 1000 patients aged 15-44 years in Brent and Avon, respectively. Of the practices in Brent that had tested for HIV, the mean annual testing rate was 0.6 (range 0.1-3.0) per 1000 patients aged 15-44 years, compared with 10.3 (range 0.4-64.9) in Avon.

### Characteristics of general practices conducting chlamydia testing

In Brent, general practices where chlamydia testing had been undertaken were larger than those that had not tested (median list size 5351 *vs. *3354, *p *= 0.04) [Table 1]. The mean age of GPs in practices that had tested for chlamydia was also younger (50.8 years *vs. *57.4 years, *p *= 0.002). The proportion of female GPs in the practice was not associated with testing. Practices that had not done tests for chlamydia tended to be in areas with higher teenage conception rates compared to those that did test (median rate 23.5 *vs. *21.6 per 1000 female population aged 15-19 years, *p *= 0.08). While there was evidence of a positive correlation between greater deprivation scores and teenage conception rates (*p *< 0.01), deprivation scores did not vary by whether or not practices had tested for chlamydia (median IMD scores 26.7 *vs. *24.6, *p *= 0.33).

In Avon, where the proportion of non-testing practices was much smaller than in Brent, no association was seen between any practice characteristic considered and practice testing status [Table 2].

**Table 2:** Characteristics of general practices in Avon, according to chlamydia testing practice

**Practice Characteristic**	**Practices that had tested for *Chlamydia trachomatis ***(n = 129)	**Practices that had not tested for *Chlamydia trachomatis ***(n = 14)	**p-value for difference**
Median list size (range)	7199 (1035-21,239)	7264 (1164-10,863)	0.44
Median IMD score	15.6	21.7	0.34
Median teen conception rate	7.6	11.6	0.58
Ratio of female GPs per 1000 female practice population (range)	0.61 (0-1.54)	0.48 (0-1.76)	0.91
Ratio of all GPs per 1000 female practice population (range)	1.3 (0.38-2.5)	1.2 (0.5-2.23)	0.93
*% GPs >50 years*	30.2	43.7	0.2

Note: Data for HIV testing not presented because only three practices did not do any HIV testing

### Characteristics of general practices conducting HIV testing

Brent practices that had not tested for HIV (other than antenatal screening tests) were of similar size and had a similar proportion of female GPs to those that had tested. As for chlamydia testing, GPs in practices that had tested for HIV tended to be younger (50.1 years *vs. *54.5 years, *p *= 0.02) (Table 1). In contrast to chlamydia testing, Brent practices that had not tested for HIV tended to be in wards with higher teenage conception rates (median 23.5 *vs. *17.4, *p *= 0.07) and in more deprived areas (median IMD score 27.1 *vs. *21.8, *p *= 0.05). Very few practices (n = 3) in Avon had not undertaken HIV tests so this analysis is not undertaken for that location.

## Discussion

Our study has demonstrated differences in chlamydia and HIV testing rates between the two study areas. Less than half of residents in the London-based study area (Brent) were registered with general practices that had tested for chlamydia and HIV, in contrast to over 90% of the non-London study area (Avon). Indeed, we found that the specialist GUM clinic in Brent conducted more chlamydia and HIV testing than all the Brent general practices combined. The historical emphasis in the UK on GUM clinics providing HIV testing services and care may explain why most testing in Brent is conducted in the GUM clinic. Greater GUM provision in London, along with a higher number of full time equivalent GPs per 1000 population in Avon (0.64 in Avon compared to 0.47 in Brent) might partly explain the more widespread testing in Avon, and the differences in general practice to GUM clinic ratios between Brent and Avon. More work is needed to understand and address these geographical differences.

Characteristics of GPs have previously been reported to be associated with levels of sexual health service provision. In our study, practices that had tested tended to have younger GPs than practices that had not tested, at least in Brent. Younger GPs may be more likely to have had education about STIs and HIV in their vocational training and consequently feel more comfortable discussing sexual health issues. This finding has important implications for the provision of effective sexual health services in primary care, and particularly the challenge of general practice participation in the NCSP, which has been identified as a key requirement for achieving the goal of controlling *Chlamydia trachomatis *infection [13]. While an association between younger practitioner age and chlamydia testing has also been observed in a study in Australia, we did not reproduce that study's association between chlamydia diagnoses and female practitioners [21]. Our findings were closer to an earlier UK study which suggested that the number of GPs per unit population was more important [10].

In both study areas, most chlamydia testing in general practice was carried out in female patients aged over 25 years, who are known to be at lower risk [22], while men received only a small proportion of chlamydia and HIV tests. These findings are consistent with a study using a large national UK primary care database, which concluded that chlamydia testing in general practice disproportionately targets women aged over 24 years and there are extremely low testing rates in men [23]. Whilst more men who have sex with men attend GUM clinics than general practice, this would not be large enough to account for the differences in testing ratios in women compared to men. We did not, however, have data to examine this formally. The disparity is more likely to reflect the routine testing of male patients for chlamydia in GUM clinics. It was worrying that there was some evidence that areas of Brent with higher teenage conception rates were more likely to have general practices that had not taken either chlamydia or HIV tests, while more deprived areas were found to have practices that had never conducted any tests for HIV. It seems therefore that STI testing in general practice continues to be limited, highly variable and poorly targeted eight years after the publication of England's National Strategy for Sexual Health and HIV [1].

A major strength of our study was the comparison of data from both general practices and GUM clinics for two contrasting populations. While linkage of information about tests and test results to area level factors such as deprivation and teenage pregnancy enabled exploration of testing in relation to hypothesised population-level indicators of sexual ill-health. However, we were not able to explore individual-level demographics in this study such as patient's ethnicity or patient-level measures of deprivation. Another possible limitation of our study is that the Brent general practices included in this analysis were those that submit samples to the Northwick Park Laboratory and so exclude seven practices in south Brent that send samples to a different laboratory. Patterns of chlamydia and HIV testing in these practices may differ, but we anticipate that general practice:GUM clinic testing ratios are likely to be similar since patients resident in this area of Brent are also closer to another GUM clinic.

Future chlamydia testing patterns will be heavily influenced by the widening implementation of opportunistic chlamydia screening in England, which is targeted at sexually active women and men under 25 years [13] and was a core proposal of the National Strategy for Sexual Health and HIV [1]. Whilst it has been recommended that chlamydia screening be integrated with other sexual health services, offering HIV testing at the same time as chlamydia testing in general practice might be challenging. Recently published HIV testing guidelines propose a strategy of active HIV testing in general practice, particularly at the time of GP registration in areas of high prevalence, in addition to targeting to "indicator diseases" (in which HIV is more common) in all populations [9]. This strategy could be considered in Brent, which has a higher prevalence of HIV than Avon, and where a much higher proportion of GPs are not yet undertaking HIV testing at all. The development of interventions to support primary care practitioners in delivering STI and HIV testing should take into account the observation that rates of testing might be low in areas with high levels of need for sexual health care. We are completing further research to develop a web-based tool to support sexual health care for people presenting to general practices in Brent and Avon, which has been informed by the findings of this study.

## Conclusions

Effective STI and HIV testing and surveillance in general practice are needed to improve sexual health care. The collection of data from the two populations in this study suggests that similar data could be collected, and our analyses applied to other localities. Such analyses could be used to develop specific strategies and innovative approaches to ensure wider availability of STI and HIV testing in young adult populations in order to identify and treat infections and contribute to reducing transmission [9]. This study suggests that the use of routinely-collected laboratory, practice-level and demographic data for monitoring sexual health service provision and informing service planning should be more widely evaluated.

## Competing interests

The authors declare that they have no competing interests.

## Authors' contributions

NL, JC, GB, SS, HM and PH designed the CaPSTI study and obtained funding. LS initiated the study and KS co-ordinated it. SS enabled access to the laboratory data and AI extracted the tables. KS analysed the data, with statistical support from CM. KS wrote the first draft and coordinated subsequent revisions of the paper. All authors contributed to multiple drafts and to the final version of this paper.

## Pre-publication history

The pre-publication history for this paper can be accessed here:

http://www.biomedcentral.com/1471-2458/10/667/prepub
